# Predictors of onset of cannabis and other drug use in male young adults: results from a longitudinal study

**DOI:** 10.1186/1471-2458-14-1202

**Published:** 2014-11-22

**Authors:** Severin Haug, Carla López Núñez, Julia Becker, Gerhard Gmel, Michael P Schaub

**Affiliations:** Swiss Research Institute for Public Health and Addiction at Zurich University, Konradstrasse 32, P.O. Box, CH - 8031, Zurich, Switzerland; Group on Addictive Behaviors, Deparment of Psychology, University of Oviedo, Oviedo, Spain; Addiction Switzerland, Lausanne, Switzerland; Alcohol Treatment Centre, Lausanne University Hospital, Lausanne, Switzerland

**Keywords:** Onset, Cannabis, Drug use, Male, Young adults

## Abstract

**Background:**

The use of cannabis and other illegal drugs is particularly prevalent in male young adults and is associated with severe health problems. This longitudinal study explored variables associated with the onset of cannabis use and the onset of illegal drug use other than cannabis separately in male young adults, including demographics, religion and religiosity, health, social context, substance use, and personality. Furthermore, we explored how far the gateway hypothesis and the common liability to addiction model are in line with the resulting prediction models.

**Methods:**

The data were gathered within the Cohort Study on Substance Use Risk Factors (C-SURF). Young men aged around 20 years provided demographic, social, health, substance use, and personality-related data at baseline. Onset of cannabis and other drug use were assessed at 15-months follow-up. Samples of 2,774 and 4,254 individuals who indicated at baseline that they have not used cannabis and other drugs, respectively, in their life and who provided follow-up data were used for the prediction models. Hierarchical logistic stepwise regressions were conducted, in order to identify predictors of the late onset of cannabis and other drug use separately.

**Results:**

Not providing for oneself, having siblings, depressiveness, parental divorce, lower parental knowledge of peers and the whereabouts, peer pressure, very low nicotine dependence, and sensation seeking were positively associated with the onset of cannabis use. Practising religion was negatively associated with the onset of cannabis use. Onset of drug use other than cannabis showed a positive association with depressiveness, antisocial personality disorder, lower parental knowledge of peers and the whereabouts, psychiatric problems of peers, problematic cannabis use, and sensation seeking.

**Conclusions:**

Consideration of the predictor variables identified within this study may help to identify young male adults for whom preventive measures for cannabis or other drug use are most appropriate. The results provide evidence for both the gateway hypothesis and the common liability to addiction model and point to further variables like depressiveness or practising of religion that might influence the onset of drug use.

## Background

Approximately 230 million individuals of the world’s adult population between the ages of 15 and 64 use an illegal drug at least once a year and about 27 million male and female adults are problem drug users; the most frequent substances consumed include cannabis, amphetamines, or ecstasy, followed by cocaine and opiates [1, 2]. Throughout the world, the use of drugs such as cannabis, cocaine, amphetamines, and opiates is more prevalent among males than females [3]. Drug consumption undermines economic and social development and contributes to crime, insecurity, and the increase of severe health problems, such as the spread of HIV [2].

The onset and escalation of drug use is associated with multiple demographic, social, psychological, personality, and family related factors [4–6]. Psychological and psychiatric problems, such as early conduct problems or conduct disorder symptoms, antisocial behaviour, depression, or anxiety [7, 8], and childhood disruptive disorders, such as attention deficit/hyperactivity disorder [9], are associated with the onset of cannabis and other drug use. Furthermore, deficits in the ability to modulate emotions or behaviours when dealing with stress have been found to be related to the initiation of drug use [10]. Personality traits, such as sensation seeking or impulsivity [11], have also been shown to have a strong relationship with the onset of substance use. Beyond psychological variables, the social and family environments are important for predicting subsequent initiation of drug consumption; these factors include the parents’ marital circumstances, a family history of drugs use, parental antisocial behaviour, ineffective communication, or poor sibling and mother–child relationships [4, 12–14]. Other factors associated with the onset and maintenance of drug use are peer pressure, academic failure, or truancy, as well as the use of other substances, such as alcohol or tobacco [5, 12, 15]. Although cannabis use in adolescence can increase the risk for the onset of other drug use [16–18], the differentiation between the onset of cannabis and other drug use was rarely made in risk factor analyses. This might be especially important in the case of Switzerland, where cannabis is rather widespread compared to other illicit drug use in young males [19].

Knowledge of the neurobiological, social, or behavioural factors associated with the initiation of drug use has increased over previous years [20]. Traditionally, such predictors have been carefully analysed in adolescents, while minimal research has focused on data concerning the onset of cannabis and other illegal drug use in adulthood. Most studies have analysed the early onset of drug consumption, that is, onset of use between early and late adolescence. However, the initiation of drug use could occur later in life, and for illegal drugs, this risk persists well beyond adolescence [12, 14, 20, 21]. Later onset of cannabis and other drug use, that is, onset of use at adulthood, is a fairly unique phenomenon and little is known of its aetiology [12]. Thus, studies focusing on the onset of drug consumption during adulthood are needed in order to identify those specific risk factors for young adults which could determine the design of preventive measures tailored to this population group.

The *gateway hypothesis* [22] has been one of the most commonly accepted theories explaining cannabis and other illegal drug use behaviour. It assumes a hierarchy in drug use with the use of legal substances (i.e. alcohol and tobacco) followed by cannabis and ‘hard’ drugs, such as cocaine or heroin. It describes the progression from legal substance use to illegal drug use, but does not highlight the specific factors associated with the risk of cannabis [23] or other drug use. The *common liability to addiction model* adopts a prudent scientific approach focusing on the common liability processes that delineate the substance-specific risk factors for use [24]. This model emphasises that a complement of psychological characteristics is related to the risk for all SUD categories and a lifestyle characterised by deviant socialisation leads to substance use initiation as one manifestation of non-conformance [25]. Thus, biobehavioural processes congenerous to all substance use disorders (SUD) categories and via interaction with multiple facets of the environment, predispose to consumption of substances, leading to SUD. In this framework, the ‘gateway’ order of drug use onset is defined opportunistically by substance availability [23].

Previous studies have mainly focused on testing hypotheses derived from these two models or on investigating the demographic, health, personality, or context-related variables associated with the onset of cannabis and other drug use. Previous research highlighted that prevention of substance use and misuse should aim at different targets related to these domains [5, 11]. However, only a few studies have explored a comprehensive set of predictors including variables from all domains [5, 11, 20], and none of the existing studies focused on the identification of the most important variables predicting later-onset cannabis and other drug use, that is, onset of use at young adulthood.

Our longitudinal study aimed at investigating the association of a comprehensive set of major risk factors for substance use derived from previous studies with the onset of cannabis and the onset of other illegal drug use separately in male young adults. The results of the study might (1) help to identify individuals for whom preventive measures for cannabis or illegal drug use are most appropriate and (2) to explore how far the current models of cannabis and other illegal drug use and their hypothesised risk factors are in line with the resulting prediction models.

## Methods

### Enrolment procedure

The present data were gathered within the Cohort Study on Substance Use Risk Factors (C-SURF), a longitudinal study designed to assess substance use patterns and their related consequences in young Swiss men approximately 20 years old. The Ethics Committee for Clinical Research of Lausanne University Medical School approved the study (Protocol No. 15/07). Study participants were enrolled in three army recruitment centres. These centres cover 21 of the 26 Swiss cantons, including all French-speaking cantons. Although the study is not fully representative, it covers mainly rural and mainly urban cantons, cantons in the north, south, west, and east of Switzerland, and the two main linguistic regions. There may be differences between individuals enrolled in different centres, but these differences reflect differences across Switzerland. The total sample was therefore analysed as a sample reflecting the two main linguistic regions. Switzerland has a mandatory army recruitment process: virtually all young men are contacted at approximately 19 years of age for determination of their eligibility for military or civil service. Thus, it is important to note that individuals who were finally selected to serve in the army were not the only ones who were enrolled in the study, as almost all young Swiss men have to go through the assessment procedures. As there is no preselection in army conscription, a virtually complete census of the Swiss male population from 21 cantons in this age group was eligible for inclusion in the study. Those who do not have to go through army assessment procedures wherein they are diagnosed with, for example, severe and chronic mental and physical disablement (e.g. trisomy 21, being blind, or being paraplegic) were ineligible. According to information from the Army, this includes less than 3% of the Swiss male population at this age.

### Participants

Enrolment in the study took place between August 2010 and November 2011. A total of 15,074 young men visited the recruitment centres. Among them, 1,829 (12.1%) did not meet the research staff because they were sick (but not chronically ill) and went home earlier. These people would have to come back to pass the recruitment procedures and would be included then. Other persons visiting the centres did not meet the research staff because they were randomly selected to participate in another study [26], or were not informed about the study by the military staff. These non-inclusions were thus mainly random and should not have influenced the findings. Of the 13,245 conscripts informed about the study, 7,563 individuals (57.1%) gave written consent to participate. Of these, 5,990 individuals (79.2%) completed the baseline questionnaire. These were sent out to them privately, two weeks after recruitment centre visits. The follow-up questionnaire was sent out to the participants approximately 15 months after the baseline questionnaire. It consisted of similar questions as the baseline questionnaire, addressing the domains ‘demography’, ‘health’, ‘social context’, ‘substance use’, ‘personality’, and ‘sexuality’. Additionally, the follow-up assessment included questions on the attendance of military training school.

For the analysis of onset of cannabis use within this study, we excluded 2,850 of the 5,990 individuals (47.6%) due to a lifetime cannabis use at baseline and an additional 366 individuals (6.1%) due to missing follow-up data. This resulted in a final sample of 2,774 individuals for the analyses of cannabis onset. For the analysis of the late onset of drug use other than cannabis, we excluded 1,017 of the 5,990 individuals (17.0%) due to a lifetime use of illegal drugs other than cannabis at baseline and an additional 719 individuals (12.0%) with missing follow-up data. This resulted in a final sample of 4,254 individuals for the analyses of the onset of use of drugs other than cannabis.

### Measures

#### Outcome variables

The onset of cannabis use at follow-up was assessed by the question ‘Did you use cannabis within the previous 12 months?’ with the response options of ‘yes’ and ‘no’. The onset of illegal drug use other than cannabis at follow-up was assessed by a series of questions to measure the frequency of use of the following 15 drugs within the previous 12 months: (1) magic mushrooms, (2) other hallucinogens, (3) salvia divinorum, (4) speed, (5) amphetamines, (6) crystal meth (Ice), (7) poppers, (8) inhalants, (9) ecstasy, (10) cocaine, crack, freebase, (11) heroin, (12) ketamine, (13) gamma-hydroxybutyrate (GHB) or gamma-butyrolactone (GBL), (14) research chemicals (e.g. mephedrone, butylone, and methedrone), and (15) spices or similar products. An onset of illegal drug use was defined as having used at least one of these 15 substances at least once within the previous 12 months.

#### Predictor variables

**Demographics** Demographic predictors were age in years and the highest completed level of education divided into two categories: a lower educational level including compulsory education or vocational school training, and a higher educational level including upper secondary education, and college and university degrees. Additional demographic predictors were the housing situation (living alone, living with parent or parents, living with partner, living with friends, or in an institution), the means of subsistence (own person, own person and other persons or institutions, other persons or institutions), living in a partnership (yes/no), and the number of siblings.

**Religion and religiosity** Religious denomination was assessed by the question ‘What is your religion (even if you do not practise or believe in God)?’ with nine response categories that we merged into the following four categories: Christian religion, Muslim religion, other religion, and no religion. Religious self-description, that is, the first question of the Religious Background and Behavior Questionnaire (RBB) [27], was used to measure religiosity. Participants were asked to indicate which of the following five categories described them best: (1) ‘I believe in God and practise religion’, (2) ‘I believe in God but do not practise religion’, (3) ‘I do not know what to believe about God’, (4) ‘I believe we cannot really know about God’ (Agnostic), (5) ‘I do not believe in God’ (Atheist).

**Health** Physical and mental health were measured by the Physical Component Summary and the Mental Component Summary of the 12-Item Short-Form Health Survey (SF-12) [28], the Major Depression Inventory (MDI) [29], and the International Physical Activity Questionnaire (IPAQ) [30]. Correlations of both the Mental and the Physical Component Summary measures of the SF-12 and the SF-36 [31], in a study using data from 9 different countries, were between .94 and .97. Various studies have shown that the SF-36 is a valid and reliable measure of population health [32]. A study of the psychometric properties of the MDI indicated adequate internal and external validity (high correlation of 0.86 with the Hamilton Depression Scale) [29]. The presence of an antisocial personality disorder (ASPD) was assessed by questions of the Mini International Neuropsychiatric Interview, which showed acceptable test-retest and inter-rater reliability [33]. It involves 2 sections with 6 childhood criteria. If 2 of these were positive, then subjects were asked about 6 behaviours since the age of 15. Three affirmative answers qualify for ASPD. Screening for an adult attention deficit syndrome was performed using a previously developed instrument [34].

**Social context** The parental situation (biological parents, adoption, etc.) was assessed by a question derived from the Alcohol Use Disorder and Associated Disabilities Interview Schedule-IV (AUDADIS-IV) [35]. The educational level of the parents was assessed and categorised analogous to the educational level of the study participant (see above). The financial situation of the family was assessed by a question from the European School Survey Project on Alcohol and Other Drugs (ESPAD) [36] which assesses, on a 7-point scale (very much below average to very much above average), how well-off an individual’s family is compared to other Swiss families.

Parenting was assessed by three variables derived from the ESPAD. The participants’ retrospective satisfaction with the relationship they had with their parents before the age of 18 was derived from two questions: ‘Before you were 18 years old, how satisfied were you usually with your relationship with (a) your mother and (b) your father?’ The responses were given on 5-point scales ranging from 1 (‘very satisfied’) to 5 (‘not satisfied at all’). The participants’ satisfaction with the relationship they had with their parents was then dichotomised at the median of the mean of the items assessing maternal and paternal relationships. Two questions were used to derive, retrospectively, parental regulation at the age of 15 years: ‘My parents set definite rules about what I was allowed to do (a) at home and (b) outside the home’. These two questions were scored on 5-point scales ranging from 1 (‘almost always’) to 5 (‘almost never’). The scores were then averaged and dichotomised at the median to obtain the parental regulation variable. Retrospective assessment of parental knowledge of peers and the whereabouts at the age of 15 years was derived by averaging and dichotomising the scores obtained from the responses to two 5-point items: ‘My parents knew (a) whom I was with, and (b) where I was in the evenings’. Parental rule setting and knowledge of peers and the whereabouts was asked at around age 15, because this is the time when peer influences become stronger, and particularly meeting with friends without the participation of parents increases [37]. For example, the Study on Health Behaviour in School-Aged Children across 41 European countries showed that peer influences, such as being four or more days per week out with friends, increase strongly between the ages of 11 and 15 [38].

The lifetime prevalence of a psychiatric, alcohol, or drug problem of the parents that demanded treatment was assessed separately for the father and the mother. The participants indicated whether a significant problem requiring treatment existed using 4 response categories (psychiatric problem, alcohol problem, drug problem, and none of these problems). A similar question addressed the previous significant alcohol, drug, or psychiatric problems of the participants’ peers. Peer pressure was assessed by a shortened version of the Peer Pressure Inventory (PPI) [39].

**Substance use** The lifetime use of alcohol was assessed by the question ‘Did you have at least 12 alcoholic drinks in your entire life?’ (yes/no). Alcohol use in the previous 12 months was assessed by the question ‘Did you have at least 1 alcoholic drink in the last 12 months?’ (yes/no). Participants who indicated ‘yes’ were classified by the Alcohol Use Disorders Identification Test (AUDIT-C) [40] as not at risk (score <4) or at risk drinkers (score ≥4) [41]; participants who indicated ‘no’ were classified into the category ‘no alcohol use in the previous 12 months’. Compared to other, more comprehensive screening instruments for alcohol use disorders, the AUDIT-C showed good sensitivity, specificity and positive predictive validity [41, 42].

To assess the lifetime use of any tobacco product, participants indicated whether they consumed at least the following quantity of one of the following products: (1) 50 cigarettes, (2) 10 water pipes (shisha), (3) 10 units of snus (4) 10 units of snuff, (5) 25 cigars or cigarillos, (6) 25 tobacco pipes. Cigarette smoking in the previous 12 months was assessed by the question ‘Did you smoke cigarettes in the last 12 months?’ (yes/no). Participants who indicated ‘yes’ were classified by the Fagerström Test for Nicotine Dependence (FTND) [43]. The FTND showed good test-retest correlation of .88 as well as appropriate criterion validity [44]. To have enough statistical power for the regression analyses we only divided smokers with very low nicotine dependence (FTND score: 0–2) from smokers with low, moderate, or high nicotine dependence (FTND score: 3–10). Participants who indicated ‘no’ were classified into the category ‘no cigarette use in the previous 12 months’.

The lifetime use of cannabis was assessed by asking ‘Have you ever consumed cannabis (grass, hashish, marihuana), more than just to try?’ (yes/no). Subsequent questions assessed cannabis use within the last 12 months with the Cannabis Use Disorders Identification Test (CUDIT) [45]. Although the internal consistency of the CUDIT seems appropriate (.72-.78), the predictive power of the instrument, tested in different studies, is mixed [46]. Based on the CUDIT score and the response to the question ‘Did you use cannabis within the last 12 months?’, participants were classified into one of the following three categories: (1) no use, (2) non-problematic cannabis use (CUDIT <8), (3) problematic cannabis use (CUDIT ≥8).

The lifetime use of drugs other than cannabis at baseline was assessed by a series of questions measuring the frequency of use (never, 1 to 3 times, more than 4 times) of the 15 drugs described in the section ‘outcome variables’.

**Personality** Sensation seeking was measured by the Brief Sensation Seeking Scale (BSSS-8) [47]. The BSSS showed good internal consistencies (.76 and .74) and was predictive of of other risk and protective factors [48]. Aggression/Hostility, Sociability, and Neuroticism/Anxiety were assessed by the corresponding subscales of the Zuckerman-Kuhlman Personality Questionnaire (ZKPQ) [49]. This instrument showed good psychometric and structural properties in four different languages with alpha coefficients above .70 [49].

**Attendance of military training school** At follow-up, we assessed whether the participants had (1) not yet started military training school, (2) started military training school, (3) finished military training school, or (4) discontinued military training school. The duration of the military training school in Switzerland is 18 or 21 weeks.

### Analysis

We initially performed separate logistic regression analyses (subsequently termed ‘univariate analyses’) to evaluate the ability of each baseline variable to predict (1) the onset of cannabis use and (2) the onset of drug use other than cannabis. To reduce multicollinearity within the final multivariate models, we subsequently developed separate multivariate prediction models for each of the following categories of predictor variables: (1) demographics, (2) religion and spirituality, (3) health, (4) social context, (5) substance use, and (6) personality. Variable selection comprised the following steps: (1) significant predictors from the univariate analyses were entered into the preliminary multivariate model. (2) Variables that were not significant were removed one by one; variables with the highest p-values were removed first (backward selection). (3) To account for suppressor effects, the resulting model was verified by tentatively adding the aforementioned excluded variables separately to the regression model. Only significant variables were retained in the separate multivariate models for each category of predictor variables (forward selection). Based on the results of these initial multivariate prediction models for each category of predictor variables, we developed overall multivariate prediction models for the onset of cannabis use and the onset of other drug use. Variable selection was conducted in an analogous way as described above, with the exception of including at step (1) all significant predictors from the separate category-specific models. Nagelkerke’s *R*^*2*^ was calculated as a goodness-of-fit measure for all multivariate models. All analyses were performed using SPSS, version 18, and *p* <0.05 was set as the significance level.

## Results

### Sample characteristics

Of the 2,774 individuals used to analyse the onset of cannabis use, 212 individuals (7.6%) had an onset within 12 months preceding the follow-up. The characteristics of the study sample for the analysis of the onset of cannabis use are depicted in Table 1. Of the 4,254 individuals used to analyse the onset of drug use other than cannabis, 222 individuals (5.2%) had an onset within 12 months preceding the follow-up. The characteristics of the study sample used to analyse the onset of other drug use are displayed in Table 2.

**Table 1:** **Characteristics of individuals with and without the onset of cannabis use and the univariate associations with the onset of cannabis use**

Variable categories and variables	No onset of cannabis use	Onset of cannabis use	***OR (95% CI)***	***p***
	n = 2,562	n = 212		
**Demographics**				
Age in years^a^, *M (SD)*	19.4 (1.2)	19.3 (1.4)	0.92 (0.82-1.05)	.21
Lower educational level (Ref.)^b^	1,892 (75.2%)	149 (72.7%)		
Higher educational level	623 (24.8%)	56 (27.3%)	1.14 (0.83-1.57)	.42
Living alone (Ref.)^c^	64 (2.5%)	6 (2.9%)		
Living with parent or parents	2,345 (92.2%)	191 (91.4%)	0.87 (0.37-2.03)	.75
Living with partner	62 (2.4%)	5 (2.4%)	0.86 (0.25-2.96)	.81
Living with friends or in institution	72 (2.8%)	7 (3.3%)	1.03 (0.33-3.25)	.95
Means of subsistence: own person (Ref.)^d^	618 (24.3%)	30 (14.4%)		
Own person and others persons or institutions	1,030 (40.6%)	83 (39.7%)	1.66 (1.08-2.55)	.02
Other persons or institutions	891 (35.1%)	96 (45.9%)	2.22 (1.46-3.39)	<.01
Not living in a partnership (Ref.)^e^	2,437 (96.0%)	198 (94.7%)		
Living in a partnership	102 (4.0%)	11 (5.3%)	1.33 (0.70-2.51)	.39
Having no siblings (Ref.)^f^	164 (6.6%)	5 (2.5%)		
One or two siblings	1,873 (75.6%)	163 (81.5%)	2.85 (1.16-7.05)	.02
Three or more siblings	440 (17.8%)	32 (16.0%)	2.38 (0.91-6.23)	.08
**Religion and religiosity**				
Christian religion (Ref.)^g^	1,929 (76.5%)	161 (78.2%)		
Muslim religion	123 (4.9%)	6 (2.9%)	0.58 (0.25-1.35)	.21
Other religion	54 (2.1%)	7 (3.4%)	1.55 (0.70-3.47)	.28
No religion	415 (16.5%)	32 (15.5%)	0.92 (0.62-1.37)	.69
Atheist (Ref.)^h^	611 (24.2%)	61 (29.6%)		
Agnostic	387 (15.3%)	29 (14.1%)	0.75 (0.47-1.19)	.22
Unsure what to think about god	304 (12.0%)	31 (15.0%)	1.02 (0.65-1.61)	.93
Belief in god but not practicing	808 (32.0%)	67 (32.5%)	0.83 (0.58-1.19)	.32
Belief in god and practicing	413 (16.4%)	18 (8.7%)	0.44 (0.25-0.75)	<.01
**Health**				
Physical health (SF-12, scale 0-100)^i^, *M (SD)*	55.0 (5.1)	55.1 (5.3)	1.00 (0.98-1.03)	.77
Mental health (SF-12, scale 0-100)^j^, *M (SD)*	50.5 (8.3)	49.3 (7.7)	0.98 (0.97-1.00)	.04
Depression (MDI, scale 0-50)^k^, *M (SD)*	6.1 (6.8)	7.7 (7.5)	1.03 (1.01-1.05)	<.01
Low physical activity (IPAQ) (Ref.)^l^	249 (10.6%)	16 (7.9%)		
Moderate physical activity	583 (24.9%)	56 (27.7%)	1.50 (0.84-2.66)	.17
High physical activity	1,510 (64.5%)	130 (64.4%)	1.34 (0.78-2.29)	.29
No attention deficit syndrome (ASRS) (Ref.)^m^	2,494 (97.5%)	201 (95.3%)		
Attention deficit syndrome	65 (2.5%)	10 (4.7%)	1.91 (0.97-3.77)	.06
No anti-social personality disorder (Ref.)^n^	2,317 (91.4%)	174 (84.1%)		
Anti-social personality disorder	217 (8.6%)	33 (15.9%)	2.03 (1.36-3.01)	<.01
**Social context**				
Grew up with both parents (Ref.)^o^	2,105 (83.1%)	155 (74.2%)		
…with parent and step-parent	101 (4.0%)	20 (9.6%)	2.69 (1.62-4.46)	<.01
…with one parent	297 (11.7%)	33 (15.8%)	1.51 (1.02-2.24)	.04
…with adoptive or foster parents or in institution	30 (1.2%)	1 (0.5%)	0.45 (0.06-3.34)	.44
No parental divorce before the age of 18 (Ref.)^p^	2,053 (81.2%)	142 (68.9%)		
Parental divorce before the age of 18	476 (18.8%)	64 (31.1%)	1.94 (1.42-2.65)	<.01
Lower educational level of the father (Ref.)^q^	1,419 (56.3%)	99 (46.7%)		
Higher educational level of the father	1,103 (43.1%)	108 (50.9%)	1.40 (1.06-1.86)	.02
Lower educational level of the mother (Ref.)^r^	1,559 (62.0%)	110 (52.9%)		
Higher educational level of the mother	956 (38.0%)	98 (47.1%)	1.45 (1.09-1.93)	.01
Financial situation of the family (Scale 1-7)^s^, *M (SD)*	3.6 (1.0)	3.5 (1.0)	0.92 (0.79-1.06)	.22
Good relationship with parents before the age of 18 (Ref.)^t^	2,115 (82.8%)	166 (78.3%)		
Bad relationship with parents before the age of 18	438 (17.2%)	46 (21.7%)	1.34 (0.95-1.88)	.10
Lower parental rule setting at the age of 15 (Ref.)^u^	972 (38.1%)	94 (44.3%)		
Higher parental rule setting at the age of 15	1,579 (61.9%)	118 (55.7%)	0.77 (0.58-1.03)	.08
Lower parental knowledge of peers and the				
whereabouts at the age of 15 (Ref.)^v^	491 (19.2%)	55 (25.9%)		
Higher parental knowledge of peers and the				
whereabouts at the age of 15	2,060 (80.4%)	157 (74.1%)	0.68 (0.49-0.94)	.02
No psychiatric problem of father at age of 15 (Ref.)^w^	2,401 (94.2%)	198 (93.8%)		
Psychiatric problem of father at age of 15	147 (5.8%)	13 (6.2%)	1.07 (0.60-1.93)	.81
No psychiatric problem of mother at age of 15 (Ref.)^x^	2,471 (97.1%)	197 (93.8%)		
Psychiatric problem of mother at age of 15	75 (2.9%)	13 (6.2%)	2.17 (1.19-3.99)	.01
No psychiatric problem of peer/s at age of 15 (Ref.)^y^	1,672 (66.1%)	124 (59.6%)		
Psychiatric problem in the peer/s at the age of 15	857 (33.9%)	84 (40.4%)	1.32 (0.99-1.76)	.06
Peer pressure (PPI total score, range -3 - +3)^z^,				
*M (SD)*	0.2 (0.4)	0.3 (0.5)	2.13 (1.47-3.08)	<.01
**Substance use**				
Never used alcohol (Ref.)^aa^	368 (15.4%)	21 (10.0%)		
Have used alcohol	2,021 (84.6%)	189 (90.0%)	1.64 (1.03-2.61)	.04
No alcohol use in the previous 12 months (Ref.)^ab^	150 (6.3%)	7 (3.4%)		
Alcohol use - not at risk (AUDIT-C)	847 (35.7%)	58 (28.0%)	1.47 (0.66-3.28)	.35
Alcohol use - at risk (AUDIT-C)	1,376 (58.0%)	142 (68.6%)	2.21 (1.02-4.81)	.04
Never used any tobacco product (Ref.)	1,692 (66.0%)	102 (48.1%)		
Have used a tobacco product	870 (34.0%)	110 (51.9%)	2.10 (1.58-2.78)	<.01
No cigarette use in the previous 12 months (Ref.)^ac^	2,037 (81.6%)	132 (65.0%)		
Very low nicotine dependence (FTND)	351 (14.1%)	63 (31.0%)	2.77 (2.01-3.82)	<.01
Low, moderate or high nicotine dependence (FTND)	109 (4.4%)	8 (3.9%)	1.13 (0.54-2.37)	.74
Never used drugs other than cannabis (Ref.)^ad^	2,429 (95.7%)	194 (92.8%)		
Used drugs other than cannabis	108 (4.3%)	15 (7.2%)	1.74 (0.99-3.04)	.05
**Personality**				
Sensation seeking (BSSS total score, range 1-5)^ae^,				
*M (SD)*	2.8 (0.8)	3.1 (0.8)	1.70 (1.43-2.02)	<.01
Aggression (ZKPQ, subscale, range 0-10)^af^, *M (SD)*	3.8 (2.2)	4.0 (2.2)	1.04 (0.98-1.11)	.24
Sociability (ZKPQ, subscale, range 0-10)^ag^, *M (SD)*	5.6 (2.3)	6.0 (2.1)	1.08 (1.01-1.15)	.02
Anxiety (ZKPQ, subscale, range 0-10)^ah^, *M (SD)*	1.9 (1.9)	2.1 (2.2)	1.08 (1.01-1.15)	.03
**Military training school**				
Not started yet (Ref.)^ai^	388 (23.6%)	34 (27.9%)		
Started	256 (15.5%)	14 (11.5%)	0.62 (0.33-1.19)	.15
Finished	897 (54.5%)	61 (50.0%)	0.78 (0.50-1.20)	.25
Discontinued	106 (6.4%)	13 (10.7%)	1.40 (0.71-2.75)	.33

Values are numbers (%) unless stated otherwise.

*Notes:* Separate binary logistic regression model for each variable. Missing values: ^a^n = 1, ^b^n = 54, ^c^n = 22, ^d^n = 26, ^e^n = 26, ^f^n = 97, ^g^n = 47, ^h^n = 45, ^i^n = 21, ^j^n = 21, ^k^n = 28, ^l^n = 230, ^m^n = 4, ^n^n = 33, ^o^n = 32, ^p^n = 39, ^q^n = 45, ^r^n = 51, ^s^n = 14, ^t^n = 9, ^u^n = 11, ^v^n = 11, ^w^n = 15, ^x^n = 18, ^y^n = 37, ^z^n = 62, ^aa^n = 175, ^ab^n = 194, ^ac^n = 74, ^ad^n = 28, ^ae^n = 3, ^af^n = 5, ^ag^n = 5, ^ah^n = 5, ^ai^n = 1,005; SF-12 = 12-Item Short-Form Health Survey; MDI = Major Depressive Inventory; IPAQ = International Physical Activity Questionnaire; AUDIT-C = Short form of the Alcohol Use Disorders Identification Test; FTND = Fagerström Test for Nicotine Dependence; ASRS = Item Screener of the Attention Deficit Syndrome Self Report Scale; BSSS = Brief Sensation Seeking Scale; ZKPQ = Zuckerman-Kuhlman Personality Questionnaire; PPI = Peer Pressure Inventory.

**Table 2:** **Characteristics of individuals with and without the onset of drug use other than cannabis and the univariate associations with the onset of drug use other than cannabis**

Variable categories and variables	No onset of drug use	Onset of drug use	***OR (95% CI)***	***p***
	n = 4,032	n = 222		
**Demographics**				
Age in years^a^, *M (SD)*	19.4 (1.2)	19.4 (1.3)	0.97 (0.87-1.09)	.64
Lower educational level (Ref.)^b^	2,911 (73.5%)	158 (72.1%)		
Higher educational level	1,048 (26.5%)	61 (27.9%)	1.07 (0.79-1.45)	.65
Living alone (Ref.)^c^	112 (2.8%)	10 (4.5%)		
Living with parent or parents	3,660 (91.4%)	193 (87.3%)	0.59 (0.30-1.15)	.12
Living with partner	92 (2.3%)	9 (4.1%)	1.10 (0.43-2.81)	.85
Living with friends or in institution	140 (3.5%)	9 (4.1%)	0.72 (0.28-1.83)	.49
Means of subsistence: own person (Ref.)^d^	879 (22.0%)	42 (19.0%)		
Own person and others persons or institutions	1,664 (41.6%)	101 (45.7%)	1.27 (0.88-1.84)	.20
Other persons or institutions	1,457 (36.4%)	78 (35.3%)	1.12 (0.76-1.65)	.56
Not living in a partnership (Ref.)^e^	3,827 (95.7%)	210 (95.0%)		
Living in a partnership	173 (4.3%)	11 (5.0%)	1.16 (0.62-2.17)	.64
Having no siblings (Ref.)^f^	253 (6.5%)	17 (7.9%)		
One or two siblings	2,965 (76.0%)	163 (75.8%)	0.82 (0.49-1.37)	.45
Three or more siblings	685 (17.6%)	35 (16.3%)	0.76 (0.42-1.38)	.37
**Religion and religiosity**				
Christian religion (Ref.)^g^	2,967 (74.5%)	162 (75.0%)		
Muslim religion	173 (4.3%)	3 (1.4%)	0.32 (0.10-1.01)	.05
Other religion	91 (2.3%)	1 (0.5%)	0.20 (0.03-1.45)	.11
No religion	751 (18.9%)	50 (23.1%)	1.22 (0.88-1.69)	.24
Atheist (Ref.)^h^	1,050 (26.4%)	72 (33.3%)		
Agnostic	676 (17.0%)	37 (17.1%)	0.80 (0.53-1.20)	.28
Unsure what to think about god	506 (12.7%)	24 (11.1%)	0.69 (0.43-1.11)	.13
Belief in god but not practicing	1,228 (30.8%)	73 (33.8%)	0.87 (0.62-1.21)	.41
Belief in god and practicing	523 (13.1%)	10 (4.6%)	0.28 (0.14-0.55)	<.01
**Health**				
Physical health (SF-12, scale 0-100)^i^, *M (SD)*	55.0 (5.1)	55.0 (5.1)	0.98 (0.96-1.01)	.20
Mental health (SF-12, scale 0-100)^j^, *M (SD)*	50.1 (8.3)	48.4 (8.6)	0.98 (0.96-0.99)	<.01
Depression (MDI, scale 0-50)^k^, *M (SD)*	6.5 (6.7)	8.2 (7.0)	1.03 (1.02-1.05)	<.01
Low physical activity (IPAQ) (Ref.)^l^	377 (10.1%)	22 (11.1%)		
Moderate physical activity	956 (25.6%)	54 (27.1%)	0.97 (0.58-1.61)	.90
High physical activity	2,402 (64.3%)	123 (61.8%)	0.88 (0.55-1.40)	.58
No attention deficit syndrome (ASRS) (Ref.)^m^	3,897 (96.9%)	207 (93.2%)		
Attention deficit syndrome	126 (3.1%)	15 (6.8%)	2.24 (1.29-3.90)	<.01
No anti-social personality disorder (Ref.)^n^	3,530 (88.2%)	160 (74.1%)		
Anti-social personality disorder	471 (11.8%)	56 (25.9%)	2.62 (1.91-3.61)	<.01
**Social context**				
Grew up with both parents (Ref.)^o^	3,228 (80.6%)	169 (77.9%)		
…with parent and step-parent	204 (5.1%)	14 (6.5%)	1.31 (0.75-2.30)	.35
…with one parent	533 (13.3%)	30 (13.8%)	1.08 (0.72-1.60)	.72
…with adoptive or foster parents or in institution	39 (1.0%)	4 (1.8%)	1.96 (0.69-5.55)	.21
No parental divorce before the age of 18 (Ref.)^p^	3,101 (77.6%)	159 (73.6%)		
Parental divorce before the age of 18	895 (22.4%)	57 (26.4%)	1.24 (0.91-1.70)	.17
Lower educational level of the father (Ref.)^q^	2,095 (52.6%)	106 (48.8%)		
Higher educational level of the father	1,887 (47.4%)	111 (51.2%)	1.16 (0.89-1.53)	.28
Lower educational level of the mother (Ref.)^r^	2,365 (59.5%)	116 (53.7%)		
Higher educational level of the mother	1,610 (40.5%)	100 (46.3%)	1.27 (0.96-1.67)	.09
Financial situation of the family (Scale 1-7)^s^, *M (SD)*	3.6 (1.0)	3.5 (1.0)	0.95 (0.83-1.09)	.44
Good relationship with parents before the age of 18 (Ref.)^t^	3,264 (81.2%)	165 (74.3%)		
Bad relationship to parents before age of 18	758 (18.8%)	57 (25.7%)	1.49 (1.09-2.03)	.01
Lower parental rule setting at the age of 15 (Ref.)^u^	1,572 (39.1%)	103 (46.4%)		
Higher parental rule setting at the age of 15	2,448 (60.9%)	119 (53.6%)	0.74 (0.57-0.97)	.03
Lower parental knowledge of peers and the				
whereabouts at the age of 15 (Ref.)^v^	899 (22.4%)	78 (35.1%)		
Higher parental knowledge of peers and the				
whereabouts at the age of 15	3,120 (77.6%)	144 (64.9%)	0.53 (0.40-0.71)	<.01
No psychiatric problem of father at age of 15 (Ref.)^w^	3,747 (93.5%)	199 (90.5%)		
Psychiatric problem of father at age of 15	261 (6.5%)	21 (9.5%)	1.52 (0.95-2.42)	.08
No psychiatric problem of mother at age of 15(Ref.)^x^	3,857 (96.2%)	207 (94.1%)		
Psychiatric problem of mother at age of 15	152 (3.8%)	13 (5.9%)	1.59 (0.89-2.86)	.12
No psychiatric problem of peer/s at age of 15 (Ref.)^y^	2,483 (62.2%)	96 (44.9%)		
Psychiatric problem of peer/s at age of 15	1,511 (37.8%)	118 (55.1%)	2.02 (1.53-2.66)	<.01
Peer pressure (PPI total score, range -3 - +3)^z^,				
*M (SD)*	0.28 (0.39)	0.39 (0.44)	2.03 (1.44-2.87)	<.01
**Substance use**				
Never used alcohol (Ref.)^aa^	438 (11.4%)	12 (5.5%)		
Have used alcohol	3,416 (88.6%)	207 (94.5%)	2.21 (1.23-3.99)	.01
No alcohol use in the previous 12 months (Ref.)^ab^	173 (4.5%)	4 (1.8%)		
Alcohol use - not at risk (AUDIT-C)	1,095 (28.6%)	29 (13.2%)	1.15 (0.40-3.30)	.80
Alcohol use - at risk (AUDIT-C)	2,563 (66.9%)	186 (84.9%)	3.14 (1.15-8.55)	.03
Never used any tobacco product (Ref.)	1,944 (48.2%)	69 (31.1%)		
Have used a tobacco product	2,089 (51.8%)	153 (68.9%)	2.06 (1.54-2.76)	<.01
No cigarette use in the previous 12 months (Ref.)^ac^	2,495 (63.9%)	91 (42.7%)		
Very low nicotine dependence (FTND)	1,048 (26.8%)	83 (39.0%)	2.17 (1.60-2.95)	<.01
Low, moderate or high nicotine dependence (FTND)	362 (9.3%)	39 (18.3%)	2.95 (2.00-4.37)	<.01
Never used cannabis (Ref.)^ad^	2,513 (62.4%)	76 (34.4%)		
Have used cannabis	1,515 (37.6%)	145 (65.6%)	3.17 (2.38-4.21)	<.01
No cannabis use in previous 12 months (Ref.)^ae^	3,204 (79.5%)	106 (47.7%)		
No problem use (CUDIT)	688 (17.1%)	86 (38.7%)	3.78 (2.81-5.08)	<.01
Problem use (CUDIT)	140 (3.5%)	30 (13.5%)	6.48 (4.18-10.05)	<.01
**Personality**				
Sensation seeking (BSSS total score, range 1-5)^af^,				
*M (SD)*	2.9 (0.8)	3.4 (0.9)	1.88 (1.59-2.24)	<.01
Aggression (ZKPQ, subscale, range 0-10)^ag^, *M (SD)*	4.0 (2.2)	4.5 (2.2)	1.10 (1.04-1.17)	<.01
Sociability (ZKPQ, subscale, range 0-10)^ah^, *M (SD)*	5.8 (2.2)	6.0 (2.1)	1.04 (0.98-1.11)	.23
Anxiety (ZKPQ, subscale, range 0-10)^ai^, *M (SD)*	1.9 (1.9)	2.2 (2.0)	1.09 (1.02-1.16)	.01
**Military training school**				
Not started yet (Ref.)^aj^	611 (24.0%)	36 (27.7%)		
Started	360 (14.1%)	12 (9.2%)	0.57 (0.29-1.10)	.09
Finished	1,405 (55.1%)	73 (56.2%)	0.88 (0.59-1.33)	.55
Discontinued	175 (6.9%)	9 (6.9%)	0.87 (0.41-1.85)	.72

Values are numbers (%) unless stated otherwise.

*Notes:* Separate binary logistic regression model for each variable. Missing values: ^a^n = 1, ^b^n = 77, ^c^n = 30, ^d^n = 34, ^e^n = 34, ^f^n = 137, ^g^n = 57, ^h^n = 56, ^i^n = 35, ^j^n = 35, ^k^n = 30, ^l^n = 321, ^m^n = 34, ^n^n = 43, ^o^n = 56, ^p^n = 64, ^q^n = 15, ^r^n = 11, ^s^n = 13, ^t^n = 14, ^u^n = 27, ^v^n = 26, ^w^n = 47, ^x^n = 182, ^y^n = 205, ^z^n = 137, ^aa^n = 6, ^ab^n = 1, ^ac^n = 10, ^ad^n = 6, ^ae^n = 9, ^af^n = 9, ^ag^n = 9, ^ah^n = 73, ^ai^n = 38, ^aj^n = 1,666; SF-12 = 12-Item Short-Form Health Survey; MDI = Major Depressive Inventory; IPAQ = International Physical Activity Questionnaire; AUDIT-C = Short form of the Alcohol Use Disorders Identification Test; FTND = Fagerström Test for Nicotine Dependence; CUDIT = Cannabis Use Disorder Identification Test; ASRS = Item Screener of the Attention Deficit Syndrome Self Report Scale; BSSS = Brief Sensation Seeking Scale; ZKPQ = Zuckerman-Kuhlman Personality Questionnaire; PPI = Peer Pressure Inventory.

### Univariate predictors of the onset of cannabis and other illegal drug use

Univariate baseline predictors of the onset of cannabis use are displayed in Table 1. Univariate predictors of the onset of other drug use are displayed in Table 2.

### Multivariate predictors of the onset of cannabis use

The separate multivariate prediction models of the onset of cannabis use for each variable category, as well as the overall prediction model, are presented in Table 3. The overall prediction model resulting from hierarchical logistic stepwise regressions (R^2^ = .11) revealed that the following variables were positively associated with the onset of cannabis use: not providing for oneself, having siblings, depressiveness, parental divorce before the age of 18, lower parental knowledge of peers and the whereabouts at the age of 15, peer pressure, low nicotine dependence, and sensation seeking. Practising religion was negatively associated with the onset of cannabis use.

**Table 3:** **Multivariate associations (using stepwise backward and forward regression** ***p*** **< .05) between participant characteristics and the onset of cannabis use**

Variable categories and variables	Separate model for each variable category		Overall model ^a,b^	
	OR (95% CI)	***p***	OR (95% CI)	***p***
**Demographics** ^**c**^				
Means of subsistence: own person (Ref.)				
Own person and others persons or institutions	1.75 (1.12-2.72)	.01	1.87 (1.16-3.00)	.01
Other persons or institutions	2.27 (1.47-3.52)	<.01	2.68 (1.66-4.34)	<.01
Having no siblings (Ref.)				
One or two siblings	2.86 (1.16-7.08)	.02	3.14 (1.24-7.95)	.02
Three or more siblings	2.53 (0.97-6.61)	.06	3.04 (1.12-8.16)	.03
**Religion and religiosity** ^**d**^				
Atheist (Ref.)				
Agnostic	0.75 (0.47-1.19)	.22	0.64 (0.39-1.06)	.09
Unsure what to think about god	1.02 (0.65-1.61)	.93	1.02 (0.63-1.65)	.94
Belief in god but not practicing	0.83 (0.58-1.19)	.32	0.78 (0.53-1.17)	.23
Belief in god and practicing	0.44 (0.25-0.75)	<.01	0.54 (0.31-0.96)	.04
**Health** ^**e**^				
Depression (MDI, scale 0–50)	1.03 (1.01-1.04)	<.01	1.03 (1.01-1.05)	<.01
No anti-social personality disorder (Ref.)				
Anti-social personality disorder	1.87 (1.25-2.80)	<.01		
**Social context** ^**f**^				
No parental divorce before the age of 18 (Ref.)				
Parental divorce before the age of 18	1.89 (1.37-2.61)	<.01	2.03 (1.44-2.87)	.04
Lower educational level of the father (Ref.)				
Higher educational level of the father	1.38 (1.03-1.85)	.03		
Lower parental knowledge of peers and the whereabouts at the age of 15 (Ref.)				
Higher parental knowledge of peers and the whereabouts at the age of 15	0.69 (0.49-0.97)	.03	0.69 (0.48-0.99)	<.01
Peer pressure (PPI total score, range -3 - +3)	2.08 (1.43-3.04)	<.01	1.56 (1.03-2.36)	.04
**Substance use** ^**g**^				
No cigarette use in the previous 12 months (Ref.)				
Very low nicotine dependence (FTND)	2.77 (2.01-3.82)	<.01	2.60 (1.83-3.69)	<.01
Low, moderate or high nicotine dependence (FTND)	1.13 (0.54-2.37)	.74	1.31 (0.60-2.84)	.50
**Personality** ^**h**^				
Sensation seeking (BSSS total score, range 1–5)	1.71 (1.44-2.04)	<.01	1.55 (1.28-1.89)	<.01
Anxiety (ZKPQ, subscale, range 0–10)	1.09 (1.02-1.17)	.02		
**Military training school**				

*Notes:* ^a^The overall model resulted from backward and forward selection of the variables included in the separate models for each variable category. ^b^ *R* ^*2*^ = .11; ^c^ *R* ^*2*^ = .02; ^d^ *R* ^*2*^ = .01; ^e^ *R* ^*2*^ = .02; ^f^ *R* ^*2*^ = .04; ^g^ *R* ^*2*^ = .03; ^h^ *R* ^*2*^ = .04. MDI = Major Depressive Inventory; FTND = Fagerström Test for Nicotine Dependence; BSSS = Brief Sensation Seeking Scale; ZKPQ = Zuckerman-Kuhlman Personality Questionnaire; PPI = Peer Pressure Inventory.

### Multivariate predictors of the onset of illegal drug use other than cannabis

The separate multivariate prediction models of the onset of other drug use for each variable category and the overall prediction model are presented in Table 4. The overall prediction model (R^2^ = .11) revealed that onset of drug use other than cannabis showed a positive association with depressiveness, the presence of an antisocial personality disorder, lower parental knowledge of peers and the whereabouts at the age of 15, psychiatric problems of peers at the age of 15, problematic cannabis use, and sensation seeking.

**Table 4:** **Multivariate associations (using stepwise backward and forward regression** ***p*** **< .05) between participant characteristics and the onset of drug use other than cannabis**

Variable categories and variables	Separate model for each variable category		Overall model ^a,b^	
	OR (95% CI)	***p***	OR (95% CI)	***p***
**Demographics**				
**Religion and religiosity** ^**c**^				
Atheist (Ref.)				
Agnostic	0.80 (0.53-1.20)	.28		
Unsure what to think about god	0.69 (0.43-1.11)	.13		
Belief in god but not practicing	0.87 (0.62-1.21)	.41		
Belief in god and practicing	0.28 (0.14-0.55)	<.01		
**Health** ^**d**^				
Depression (MDI, scale 0–50)	1.03 (1.01-1.04)	<.01	1.02 (1.00-1.04)	.02
No attention deficit syndrome (ASRS) (Ref.)				
Attention deficit syndrome	1.84 (1.04-3.27)	.04		
No anti-social personality disorder (Ref.)				
Anti-social personality disorder	2.50 (1.81-3.46)	<.01	1.51 (1.06-2.13)	.02
**Social context** ^**e**^				
Lower parental knowledge of peers and the				
whereabouts at the age of 15 (Ref.)				
Higher parental knowledge of peers and the				
whereabouts at the age of 15	0.58 (0.44-0.79)	<.01	0.73 (0.54-1.00)	.05
No psychiatric problem of peer/s at age of 15 (Ref.)				
Psychiatric problem of peer/s at age of 15	1.82 (1.37-2.42)	<.01	1.53 (1.15-2.05)	<.01
Peer pressure (PPI total score, range -3 - +3)	1.79 (1.26-2.54)	<.01		
**Substance use** ^**f**^				
No cigarette use in the previous 12 months (Ref.)				
Very low nicotine dependence (FTND)	1.19 (0.84-1.69)	.32		
Low, moderate or high nicotine dependence (FTND)	1.58 (1.02-2.43)	.04		
No cannabis use in the previous 12 months (Ref.)^ab^				
Low cannabis dependence (CUDIT)	3.92 (2.90-5.31)	<.01	3.01 (2.20-4.10)	<.01
Moderate or high cannabis dependence (CUDIT)	7.08 (4.54-11.03)	<.01	4.13 (2.59-6.57)	<.01
**Personality** ^**g**^				
Sensation seeking (BSSS total score, range 1–5)	1.90 (1.60-2.26)	<.01	1.49 (1.23-1.79)	<.01
Anxiety (ZKPQ, subscale, range 0–10)	1.11 (1.04-1.18)	<.01		
**Military training school**				

*Notes:* ^a^The overall model resulted from backward and forward selection of the variables included in the separate models for each variable category. ^b^ *R* ^*2*^ = .11; ^c^ *R* ^*2*^ = .01; ^d^ *R* ^*2*^ = .03; ^e^ *R* ^*2*^ = .03; ^f^ *R* ^*2*^ = .08; ^g^ *R* ^*2*^ = .04. MDI = Major Depressive Inventory; FTND = Fagerström Test for Nicotine Dependence; CUDIT = Cannabis Use Disorder Identification Test; BSSS = Brief Sensation Seeking Scale; ZKPQ = Zuckerman-Kuhlman Personality Questionnaire; PPI = Peer Pressure Inventory.

## Discussion

This longitudinal study aimed to identify predictors of the late onset of cannabis and other drug use in male young adults. The study revealed four main findings: (1) A combination of variables from several domains provides the best power to predict the onset of both cannabis and other illegal drug use. (2) The results provide evidence for both the gateway hypothesis and the common liability to addiction model. (3) Of those variables that have been studied, the following are the most relevant to predict the onset of cannabis use: the means of subsistence, the number of siblings, religiosity, depression, parental divorce before the age of 18, low parental knowledge of peers and the whereabouts, peer pressure, nicotine dependence, and sensation seeking. (4) For the onset of other drug use, depression, the presence of an antisocial personality disorder, lower parental knowledge of peers and the whereabouts, psychiatric problems of peers, cannabis use, and sensation seeking are the most relevant predictors.

Taking into account the goodness of fit of the explored variable categories, current substance use, personality, and social context variables are the most important domains for predicting onset of cannabis and drug use. This result is in line with both the gateway hypothesis [22], which assumes a hierarchy in drug use with the use of legal substances followed by cannabis and ‘hard’ drugs, and the common liability to addiction model [24], emphasising the importance of socialisation and personality characteristics. However, beyond the variables discussed in these models, health-related variables, particularly depressiveness might influence the onset of drug use.

Referring to the predictors of the onset of cannabis use, our findings mainly underline the results from previous studies. Having siblings was identified as a risk factor for substance use initiation, especially when they are older or share common characteristics with the consumer [4]. Previous research also revealed that cannabis use is a common behaviour among young adults who have problems in their means of subsistence [50]. Our study showed that religiosity, defined as believing in God and practising religion, protected from the onset of cannabis use. This is in line with previous research based on cross-sectional data showing that single dimensions of religiosity, namely, social religiosity and perceived religious support, were correlated with lower cannabis use [51]. Previous studies postulated that parental monitoring or permissiveness may be related to religiosity with higher parental supervision in families practising religiosity [52]. However, the results of our final prediction model showed that religiosity and parental knowledge of peers and the whereabouts at the age of 15 were independently related to the onset of cannabis use. The finding that parental divorce at an early age predicted the onset of cannabis use is in line with prior results showing that children who are exposed to family problems, including family disruption and conflict, are more likely to use drugs such as cannabis in both adolescence and young adulthood [4, 6]. Concerning the health-related variables, our study shows that depressiveness plays an important role in the later onset of cannabis use [7]. Sensation seeking was another strong predictor for the onset of cannabis use, which has been traditionally studied in relation to this substance [11]. Taking into account that sensation seeking usually peaks in late adolescence and then declines with age [53], it is not surprising that it also constitutes a relevant predictor of the onset of cannabis use during early adulthood. In line with the finding that young adults who exhibit high sensation seeking typically tend to seek peers with similar interests who further encourage risk-taking behaviour [54], peer pressure and sensation seeking were predictive of the onset of cannabis use. Finally, our results confirm previous studies showing that nicotine dependence predicts cannabis initiation, which is also in line with the gateway hypothesis [55].

Some variables predicting onset of cannabis use were also predictive of the onset of drug use other than cannabis, including depressiveness, religiosity, parental knowledge of peers and the whereabouts, anxiety, and sensation-seeking. These variables present promising candidates for inclusion in a common liability of addiction model. Psychopathological factors, such as a previous history of depression symptoms or anxiety, have been commonly studied as a risk factor of drug use [8]; furthermore, a more permissive parenting style was related to illegal drug experience in previous studies [4, 6]. Believing in God and practising religion also protected from the initiation of illegal drug use, suggesting that religiosity may be a protective factor against cannabis and other drug use [56]. Moreover, late adolescence and young adulthood is a period of heightened experimentation with risky behaviour; therefore, it is not surprising that sensation-seeking also plays an important role as a predictor of cannabis and other substance use [54]. Furthermore, there are some unique predictors of drug onset, including psychiatric problems of the young adults’ peers at the age of 15 and the presence of an antisocial personality disorder. The latter has been related to substance use and abuse in previous studies [57].

To date, the majority of interventions for prevention of drug use of young people are provided on the level of the community, school, or school class and are not targeted to specific population groups [58, 59]. Only few interventions are targeted to specific risk groups, mainly defined by current cigarette, alcohol or cannabis use and therefore reflecting the risk factors defined by the gateway hypothesis or are targeted to ethnic minorities or socioeconomically disadvantaged groups. Taking into account the comprehensive set of variables investigated and the result that the explained variance of 11% of the final prediction models was relatively poor, there is still justification for non-targeted interventions. Based on our results there is also justification for using variables of substance use to target intervention measures. Among the variable categories considered, previous substance use in general and cannabis use in particular, showed the best predictive value for the onset of drug use other than cannabis with *R*^*2*^ = .08 and explains the majority of the variance of the overall model. However, for the prediction of onset of cannabis use, variables of the social context (*R*^*2*^ = .04) and personality factors *(R*^*2*^ = .04) seem to be equally important as substance use (*R*^*2*^ = .03). Consequently, personality and social context variables like sensation seeking, peer pressure, or parental divorce are variables which might additionally be considered in the development of screening instruments for persons at risk for cannabis use.

The limitations of the study are (1) that only men were included, as only young men have to visit the army recruitment centres, and that the results could not be generalised to young women, (2) that the participants were observed for a relatively short time period, as they were reassessed only once after 15 months, (3) that there is a period of 3 months that is not covered by the assessments because the follow-up assessment takes into account only the preceding 12 months, (4) that all data rely on self-report without biochemical verification or inclusion of genetic risk factors, (5) that some of the instruments used have not been validated or for the sake of brevity short-forms or single items of validated instruments were used, and (6) that statistical power was low for some of the examined predictor variables, particularly within the multivariate models predicting onset of drug use other than cannabis. The variables housing situation, religion, parental situation and alcohol use in the previous 12 months were most impacted by a loss of statistical power.

The strengths of the study include that the analyses are based on a relatively large dataset of an age-homogeneous group, and the comprehensiveness of the predictor variables investigated. Further research should address whether the predictors identified are male-specific or whether they could be generalised to young male and women.

## Conclusions

The results of this study provide evidence for the gateway hypothesis and the common liability to addiction model and point to further variables, not addressed in these models, like depressiveness, that might influence the onset of drug use. Furthermore, the results may help to identify male young adults for whom preventive measures for cannabis or other drug use are most appropriate and to develop screening instruments for the identification of male young adults at risk for cannabis and other drug use.

## Abbreviations

AUDIT-C: Alcohol use disorders identification test
AUDADIS-IV: Alcohol use disorder and associated disabilities interview schedule-IV
ASPD: Anti-social personality disorder
BSSS-8: Brief sensation seeking scale
C-SURF: Cohort study on substance use risk factors
CUDIT: Cannabis use disorders identification test
ESPAD: European school survey project on alcohol and other drugs
FTND: Fagerström test for nicotine dependence
GHB: Gammahydroxybutyrate
GBL: Gammabutyrolactone
IPAQ: International physical activity questionnaire
MDI: Major depression inventory
PPI: Peer pressure inventory
RBB: Religious background and behavior questionnaire
ZKPQ: Zuckerman-Kuhlman personality questionnaire.
